# Hydraulic Strategies of Cool‐Temperate Trees and Lianas Against Freeze–Thaw Embolism: Is Cold Winter More Severe on Lianas?

**DOI:** 10.1111/pce.70889

**Published:** 2026-09-17

**Authors:** Ryuji Ichihashi, Haruhiko Taneda, Yasuhiro Utsumi

**Affiliations:** ^1^ Ashoro Research Forest, Faculty of Agriculture Kyushu University Ashoro Hokkaido Japan; ^2^ Nikko Botanical Garden, Graduate School of Science The University of Tokyo Nikko Tochigi Japan

**Keywords:** deciduous forest, diffuse porous, Japan, ring porous, root pressure, safety–efficiency trade‐off, vessel size

## Abstract

Lianas are abundant and influential in tropical forests but decline sharply in cooler regions. This pattern has been attributed to their reliance on a small number of large vessels, which are vulnerable to freeze–thaw embolism. However, the hydraulic functioning of lianas in cooler regions remains poorly understood. We examined the hydraulic properties of nine tree and four liana species in a cool‐temperate forest. Compared with tree branches of similar diameter and age, liana branches had substantially wider vessels and, on average, 10 times greater specific conductivity (*K*_s_), supporting approximately six times more leaf area. While they experienced a nearly complete loss of *K*_s_ during winter, conductivity was fully restored before or during leaf expansion. This recovery was achieved either by refilling embolised vessels using positive xylem pressure or by reconstructing the hydraulic pathway with newly formed vessels, similar to mechanisms observed in trees. Seasonal sap‐flow patterns during the growing season fell within the range observed for trees. Thus, cool‐temperate lianas maintained hydraulic function at least as effectively as trees. Our results do not support a simple freeze‐vulnerability hypothesis but instead suggest that the ecological success of cool‐temperate lianas depends strongly on mechanisms that restore stem hydraulic function after winter.

## Summary statement

Cool‐temperate lianas maintained hydraulic function as effectively as trees due to shared recovery mechanisms following declines under subfreezing temperatures; thus, the classic freeze‑vulnerability hypothesis alone cannot explain liana latitudinal distributions.

## 1 Introduction

Lianas (woody climbers) are most abundant and diverse in the tropics, where they play important roles in forest dynamics and ecosystem functioning (van der Heijden et al. 2015; Jackson et al. 2026). Their abundance declines markedly towards temperate and colder regions (Gentry 1991; Schnitzer 2005), yet temperate lianas can still exert a notable influence on the structure and ecological processes of their habitats (Ladwig and Meiners 2015; Ichihashi et al. 2017; Peters et al. 2023). This pronounced latitudinal pattern has generally been explained by the hypothesis that the hydraulic architecture of lianas is poorly adapted to cold winters (Gentry 1991; Schnitzer 2005; Jiménez‐Castillo and Lusk 2013; Lobos‐Catalán and Jiménez‐Castillo 2023).

Many lianas possess characteristically wide and long vessels for water transport compared with co‐occurring trees (Gartner et al. 1990; Jacobsen et al. 2012; Schnitzer 2018). These large vessels enable efficient water transport, allowing lianas to supply water to disproportionately large canopies through their slender stems (Ichihashi et al. 2017). However, larger vessels are generally more vulnerable to xylem embolism, particularly under freeze–thaw conditions (Davis et al. 1999; Hacke et al. 2017; Venturas et al. 2017; Zhang et al. 2024). When xylem sap freezes, dissolved gases are excluded from the ice phase and form bubbles within xylem conduits. Upon thawing, these bubbles may either dissolve back into the sap or expand and block water transport (i.e., embolised). Because wider conduits generate larger bubbles that are less likely to dissolve, species with wider vessels should be particularly vulnerable to freeze‐thaw‐induced embolism (Davis et al. 1999; Pittermann and Sperry 2006; Venturas et al. 2017). This vulnerability has therefore been proposed as a key mechanism limiting the ecological success of lianas in regions with cold winters.

The freeze‐vulnerability hypothesis for lianas has a notable implication under global climate change: if this hypothesis is true, the rising winter temperatures (Contosta et al. 2024) may increase the prevalence of lianas in temperate forests and allow them to have a more obvious influence on forest dynamics and ecological processes (Ladwig and Meiners 2015; Ichihashi et al. 2017; Perring et al. 2020). To date, however, only a handful of studies have examined the effects of cold winters on the hydraulic function of lianas. In evergreen forests of temperate South America, lianas experienced greater seasonal declines in stem hydraulic conductivity than co‐occurring trees during winter (Jiménez‐Castillo and Lusk 2013). A subsequent study conducted across a latitudinal gradient within the same region found that lianas inhabiting colder sites tended to exhibit smaller vessel diameters, lower stem hydraulic conductivity and reduced apical growth rates (Lobos‐Catalán and Jiménez‐Castillo 2023). Although these findings are broadly consistent with the freeze‐vulnerability hypothesis, vessel diameter in lianas from temperate Japan showed no clear relationship with mean annual temperature across sites (Kusakabe et al. 2025). Collectively, the available evidence remains limited and mixed, highlighting the need for further ecophysiological studies of temperate lianas.

Maintaining hydraulic function is also a major challenge for trees in cold environments. To cope with this challenge, trees typically employ one of three strategies. The first involves the use of narrow and short conduits that are resistant to freeze–thaw embolism (Davis et al. 1999; Pittermann and Sperry 2006; Hacke et al. 2017). The second strategy is found in species that generate substantial positive xylem pressure before budbreak, thereby refilling embolised vessels and restoring hydraulic function (Utsumi et al. 1998; Westhoff et al. 2008; Niu et al. 2017). The third strategy, known from ring‐porous trees, involves abandoning embolised vessels after winter and reconstructing the hydraulic pathway through the production of new vessels each spring (Sperry et al. 1994; Hacke and Sauter 1996; Utsumi et al. 1999). While the first strategy minimises embolism formation under freezing conditions, the latter two restore hydraulic function after winter. If lianas possess similar mechanisms for restoring hydraulic function, subfreezing temperatures may not necessarily impair their growth and survival more severely than those of trees despite their larger vessel diameters. Indeed, seasonal positive xylem pressure has been documented in several temperate lianas, including grapes (*Vitis* spp.) (Sperry et al. 1987; Tibbetts and Ewers 2000), and kiwifruit vines (*Actinidia* spp.) (Clearwater et al. 2007). Another temperate liana, *Celastrus orbiculatus*, appears to conduct water primarily through current‐year xylem, suggesting a strategy of annual hydraulic reconstruction (Tibbetts and Ewers 2000). However, it remains unclear whether and to what extent these hydraulic phenomena contribute to the overwintering strategies of temperate lianas.

In this study, we examined the hydraulic characteristics of nine tree and four liana species common in cool‐temperate forests of Japan. Evaluations included xylem anatomy, seasonal changes in stem hydraulic conductivity and sap flux, and xylem pressure profiles in early spring. We evaluated the freeze‐vulnerability hypothesis for lianas using four working hypotheses. Relative to trees, lianas are expected to (i) possess larger vessels and achieve higher stem hydraulic conductivity during summer, but (ii) experience a greater loss of conductivity during winter, resulting in (iii) delayed recovery of conductivity in spring or persistently reduced conductivity throughout the growing season and, consequently, (iv) reduced whole‐plant transpiration during the early or entire growing season. Hypothesis (iv) was evaluated using relative seasonal changes in sap flux. Consequently, the possibility of chronic reductions in transpiration throughout the growing season could not be tested directly and was instead discussed in light of complementary evidence. In parallel with these tests, we also asked whether these cool‐temperate lianas and trees adopted any of the three hydraulic strategies for coping with freeze‐thaw embolism during winter: *resistant*, *refill*, and *reconstruct* (hereafter, these words in italics are used to indicate the corresponding functional types).

## 2 Materials and Methods

### 2.1 Study Site and Species

This study was conducted in a secondary forest (32°22–23′N, 131°10′E, ca. 1050 m a.s.l.) in the Shiiba Research Forest of Kyushu University, Miyazaki, Japan. The site is in a cool‐temperate zone with high precipitation; the mean annual temperature and precipitation in the recent 5 years were 10.9°C and 4131 mm, respectively (based on the data for 2015–2017, 2020 and 2022, when complete annual data sets were available). The meteorological data for the study period are shown in Figure S1. These data were monitored at the site by the Shiiba Research Forest and are available to researchers upon request. During this period, the minimum air temperatures were approximately −15°C, and approximately 90 freeze‐thaw cycles (below and above 0°C, respectively) occurred every winter (Figure S1). The forest mainly consisted of deciduous broad‐leaved trees intermixed with conifers and contained evergreen broad‐leaved trees at a lower frequency.

The nine tree species examined (Table S1) are dominant canopy trees in this region (Ogata et al. 2017). The four stem‐twining liana species were the only common lianas around the study site that were sufficiently abundant for all measurements. Canopy leaves began to expand from late April to early May and were mostly lost by early November (Table S1). Before conducting the measurements, we assessed the extent of water‐conducting xylem (i.e., whether it was restricted to current‐year xylem) for each species by applying a dye (0.1% safranin) solution to stem segments prepared in the same manner as those used for hydraulic conductivity measurements (see below). Photographs of the stained stem sections are shown in Figure S2. It should be noted that trees relying on current‐year xylem are often associated with ring‐porous wood, whereas those relying on older xylem are commonly associated with diffuse‐porous wood. However, ring‐porous and diffuse‐porous are anatomical classifications based on vessel arrangement within growth rings rather than direct functional categories. As our study focused on xylem function rather than wood anatomy, we did not assign these classifications to the study species. This was particularly important for the lianas included in the study, in which growth‐ring structure and earlywood‐latewood differentiation were not always sufficiently clear.

### 2.2 Stem Hydraulic Conductivity

The hydraulic conductivities of distal stem segments were measured in three seasons over a 1‐year period: May (mid‐spring), August–September (mid‐summer) 2021, and February (late winter) 2022. Sample branches were collected from five adult plants (≥ 10 m in top height) growing along forest roads for each species on each measuring occasion. For lianas, all sampled plants were mature individuals that had climbed into the host‐tree canopies. The target plants were not necessarily identical throughout the three seasons, and those used for sap flow measurements (described later) were excluded. Distal branches approximately 1.5 m from the apex (for trees) or branch segments of approximately 3 m in length (for lianas) were taken from lower but unshaded canopy positions (approximately 3–5 m above the ground) of target plants in the morning and/or during the rain. The cut branches were transported to the laboratory while immersed in water in a container. During summer sampling, all leaves distal to the sample stem segments were collected.

In the laboratory, stem segments without lateral shoots, typically 1.0–1.5 cm in basal diameter, 5–12 years old, and 10–15 cm (for trees) or 30–50 cm (for lianas) in length, were cut from the centres of the sampled branches. The diameters and ages of the segments were similar between trees (1.22 ± 0.12 cm, 8.5 ± 0.3 years, mean ± SE; *n* = 135) and lianas (1.17 ± 0.28 cm, 7.8 ± 0.3 years; *n* = 60). Hydraulic conductivity was measured by connecting the segments to a tubing system where a filtered 20 mM KCl solution was passed through the stem, driven by a hydraulic head (Sperry et al. 1988; Zwieniecki et al. 2001). Assuming that liana vessels could be longer than the length of stem segments (Gartner et al. 1990; Jacobsen et al. 2012), the hydraulic head for flow rate measurements was set below 5 cm (equivalent to approximately 0.5 kPa of gravitational force), ensuring that refilling of embolised conduits ≤ 600 μm in diameter and open at both ends would theoretically not occur (Melcher et al. 2012). Specific conductivity (*K*_s_, kg s^−1^ m^−1^ MPa^−1^) was calculated as the mass flow rate divided by the pressure gradient along the segment and the xylem area. For each summer sample, the total leaf area distal to the stem segment was determined to calculate the leaf area‐specific conductivity (*K*_l_, kg s^−1^ m^−1^ MPa^−1^) and Huber value (HV, xylem area to leaf area ratio, m^2^ m^−2^).

Although the ‘maximum’ conductivity was measured after flushing the segments at 100 kPa for 15 min, only the native values were used for analyses. This was primarily because in species relying on current‐year xylem (Figure S2), the vessels in older growth rings could be refilled during flushing; as a result, *K*_s_ in August increased by up to 331% (for *Castanea crenata*) after flushing (Figure S3). This is unlikely to occur under natural conditions. In addition, because the study site experiences heavy rainfall during the growing season (Figure S1), plant hydraulics were not markedly affected by drought. For the species that were not current‐year xylem‐dependent, *K*_s_ in August did not increase appreciably after flushing (increased by a maximum of 21.1% for *Acer sieboldianum*, Figure S3); this difference should have little effect on the conclusions of the present study. Accordingly, the percentage loss of *K*_s_ (PLC) in the present study indicated a decrease in *K*_s_ in winter compared to that in summer.

### 2.3 Vessel‐Related Xylem Anatomy

During the summer, the vessel anatomy in the stem segments used for the *K*_s_ measurement was investigated. Thin transverse sections of the stems were prepared using a blade‐rotating microtome (MTH‐1; Nippon Medical & Chemical Instrument Co., Osaka, Japan). Images of these sections were captured by a digital camera through an optical microscope (×40, CX41; Olympus, Tokyo, Japan). In the microscopic images, a 3–5 mm^2^ region of outer xylem was analysed using Fiji software (Schindelin et al. 2012). We counted conduits > 80 μm^2^ in lumen area (approximately > 10 μm in diameter) as vessels, and computed the diameters (*d*) of individual vessels from lumen areas assuming that the vessels were circular. The hydraulically weighted mean vessel diameter was determined as *D*_h_ = ∑*d* ^5^/∑*d* ^4^ (Sperry et al. 1994; Davis et al. 1999) for each sample. We also determined the density of vessels accounting for 95% of the theoretical conductivity (mm^−2^) and the fraction of vessel lumen in the xylem area (mm^2^ mm^−2^).

### 2.4 Sap Flux Measurement

Sap flux was measured at the stem base of three plants of each species during the growing season (April–November) in 2020 and 2021 (each plant was measured once in either year). The selected plants were mature, ≥ 10 m in height, appeared healthy and were located along forest roads without significant shading. The thermal dissipation method developed by Granier (1985) was used for these measurements. This method estimates sap flux from the temperature difference between two probes inserted vertically into the target stem, with constant heat applied to the upper probe. The method was applied to lianas and trees in the field following the method described by Ichihashi et al. (2017). For each plant, measurements were performed using one sensor (a pair of probes), which was installed in the outermost xylem at approximately 1.3 m above the ground. We used 10‐mm probes with a 0.15‐W power supply for lianas and for trees relying on current‐year xylem, and 20‐mm‐long and 2‐mm‐diameter probes with a 0.2‐W power supply for other trees (Wullschleger and King 2000; James et al. 2002). A data logger (MCR‐4V; T&D, Matsumoto, Japan) monitored the voltage signals from the thermocouples equipped with the probes every 10 s, averaging the data over 30‐min intervals. Although researchers are generally recommended to develop their own calibration to obtain accurate estimates of flow rate (Fuchs et al. 2017; Ichihashi et al. 2017), we instead calculated provisional flux values using Granier's original calibration and analysed relative changes in flux within individual plants during the growing season.

### 2.5 Monitoring of Xylem Pressure in Early Spring

Xylem pressure was measured for three plants of each species from mid‐February to early May 2023 (but measurements were unsuccessful in several plants due to electrical issues or irretrievable leaks of sap from the holes). Sample plants were selected from healthy‐looking adult trees along forest roads, excluding those already used for sap flow measurements. A hole with a diameter of 2.5 mm and depth of 10–20 mm (depending on the thickness of the target stems) was drilled on the target stem at approximately 1 m above the ground. A hollow bolt with an outer diameter of 3.0 mm and a shaft length of either 10 mm or 15 mm was screwed into the drilled hole, ensuring it did not fill the full depth of the hole, after wrapping the sealing tape around the threads. The bolthead was connected to a pressure transducer (PX26‐30DV or 100DV; Omega Engineering, Norwalk, CT, USA) using a water‐filled tube. The voltage signal from the sensor was monitored every 10 s, and 30‐min averages were recorded using a data logger (CR 1000 or CR 1000X; Campbell Scientific, or MCR‐4V, T&D). The schematic illustrations and photographs of the measurement system are shown in Figure S4.

### 2.6 Data Analyses

All statistical analyses were conducted using R 4.4.1 (R Core Team 2024). The means for conductivity and anatomical parameters were compared among species (or among seasons within species for the analysis of the seasonal variation in *K*_s_) by ANOVA with Tukey–Kramer's multiple comparison test (*n* = 5 for each parameter within each species and season). Seasonal variations in sap flux were analysed for individual species in relation to the meteorological conditions represented by the potential evapotranspiration (PET; Penman 1948; Shuttleworth 2007). The sap flux data were initially transformed into daily values relative to the average of the 10 highest daily records over the measurement period (relative *F*_d_day_). A linear mixed model incorporating sample plants as a random factor was used to test whether PET and season (May and October relative to August) had significant effects on *F*_d_day_. *n* = 23–31, 25–31 and 24–31 plant‐month observations in May, August and October, respectively, for individual plants, due to missing data for several plants (actual data used for the analyses are presented in Data Sheet S2). For individual plants that exhibited obvious xylem pressure in early spring, the potential height of water ascent driven by xylem pressure was estimated. Estimates were based on the maximum daily mean xylem pressure, the height at which the pressure sensor was installed, and the assumption that 10 kPa of pressure can raise a water column by 1 m.

## 3 Results

### 3.1 Test of Hypothesis (I): Vessel Size and Stem Hydraulic Conductivity During Summer

Lianas generally had wide vessels; the hydraulically weighted mean diameter (*D*_h_) exceeded 150 μm in every species and was, on average, more than three times that of trees (Figure 1a, Table S2). The wide individual vessels of lianas were associated with a smaller vessel density (approximately one‐ninth, on average, Table S2, Figure S4a), although overall porosity remained higher in liana stems (the fraction of vessel lumen in xylem area was approximately 0.3) than in trees (0.1–0.2) (Table S2, Figure S4b). Thus, lianas relied on a smaller number of characteristically wider vessels for water transport.

**Figure 1:**
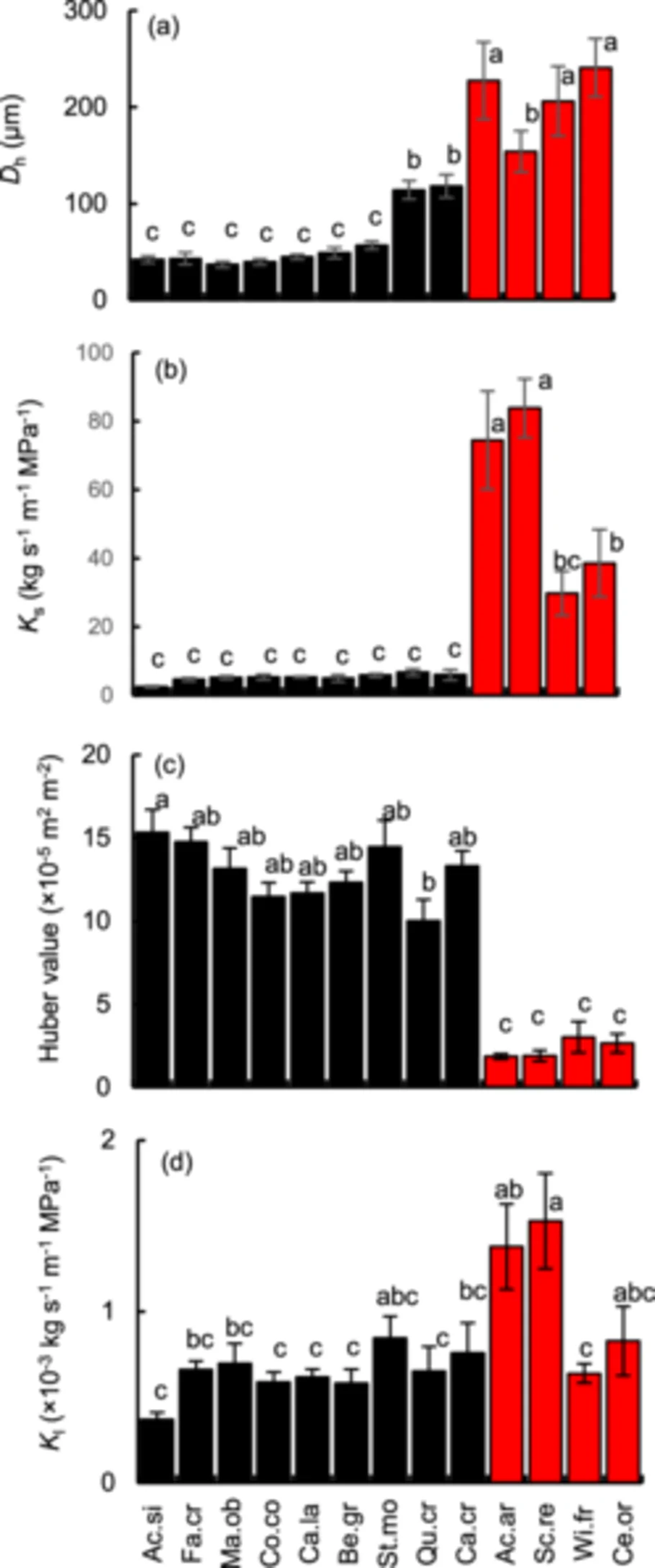
Hydraulically weighted mean vessel diameter (*D*_h_, a), xylem area‐specific conductivity (*K*_s_, b), Huber value (sapwood‐to‐leaf area ratio, c) and leaf area‐specific conductivity (*K*_l_, d) of distal stem segments of cool‐temperate trees (black) and lianas (red) measured in summer. Species names are abbreviated using the first two letters of both the genus and specific epithet. Mean values were compared among species using one‐way ANOVA followed by Tukey–Kramer's multiple comparison test; different letters above the bars indicate significant differences (*p* < 0.05). Error bars indicate standard errors. *n* = 5 for each species.

Consistent with their large vessel size, xylem area‐specific conductivity (*K*_s_) measured in summer was, on average, 10 times greater in liana stems than in trees (Figure 1b, Table S2). Because liana branches accommodated six times more leaf area per unit basal sapwood area (i.e., the reciprocal of Huber values, Figure 1c, Table S2), conductivity became more comparable between lianas and trees when evaluated on a leaf‐area basis (*K*_l_, Figure 1d, Table S2). Thus, in the middle of the growing season, the *K*_s_ of liana stems was sufficiently high to supply water to many times larger leaf areas than that of tree stems, which supported the hypothesis (i).

### 3.2 Test of Hypotheses (II) and (III): Winter Loss and Seasonal Recovery of Stem Hydraulic Conductivity

The *K*_s_ declined by late winter to varying degrees among species, ranging from no significant decline in four tree species to nearly complete loss (Figure 2). All liana species exhibited an almost complete loss of *K*_s_. These results were consistent with hypothesis (ii), although near‐complete loss of conductivity was not unique to lianas and also occurred in several tree species.

**Figure 2:**
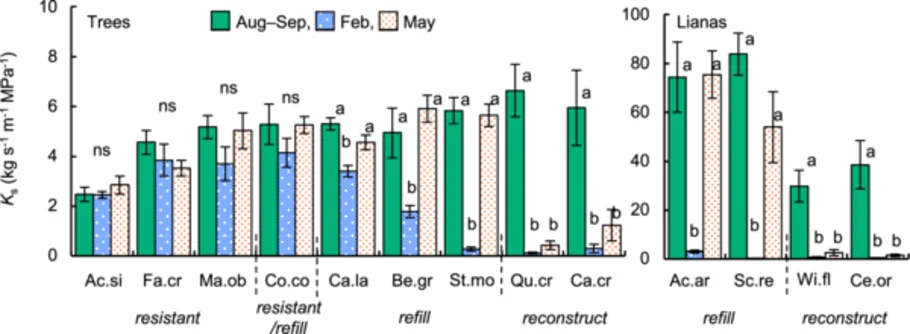
Xylem area‐specific conductivity (*K*_s_) of distal stem segments of cool‐temperate trees and lianas measured in summer (August–September), winter (February) and spring (May). Species names are abbreviated using the first two letters of both the genus and specific epithet. Species are grouped according to their strategies against freeze‐thaw embolism (see text). Seasonal differences within species were tested using one‐way ANOVA followed by Tukey–Kramer's multiple comparison test; different letters above the bars indicate significant differences (*p* < 0.05). Error bars indicate standard errors. *n* = 5 for each bar. [Color figure can be viewed at wileyonlinelibrary.com]

Among the nine species in which *K*_s_ declined during winter, conductivity had recovered by May, when the new growing season began, in five species, including two lianas (Figure 2). In contrast, no substantial recovery was observed in the remaining two tree and two liana species. Thus, delayed recovery of conductivity was not a common feature of lianas but was characteristic of certain species of both lianas and trees. The possibility of a chronic reduction in *K*_s_ throughout the growing season could be rejected based on the high summer *K*_s_ observed in liana stems (Figure 1b), which did not increase significantly after experimental refilling (except in the current‐year‐xylem‐dependent species, *C. orbiculatus*, Figure S3). Overall, the present results did not support hypothesis (iii).

### 3.3 Test of Hypothesis (IV): Seasonal Changes in Whole‐Plant Transpiration

The relationship between daily sap flux (*F*_d_day_) and PET varied seasonally in May, August, and October in most tree and liana species (Figure 3). *F*_d_day_ was lower at a given PET in May than in August, indicating less active transpiration in the early growing season, for several species of trees and lianas, including those that exhibited little recovery of stem *K*_s_ by May (i.e., *Q. crispula* and *C. crenata* among trees, and *W. floribunda* and *C. orbiculatus* among lianas; Figure 2). In contrast, sap flow was more active in May than in August in the tree species *C. controversa* and the liana species *S. repanda*. Overall, the seasonal profiles of transpiration activity appeared to be species‐specific, and any particular trend common to lianas, such as a stagnation during the early growing season, was not observed. Thus, except for the possibility of persistent stagnation throughout the entire growing season, the hypothesis (iv) was not supported by the present results.

**Figure 3:**
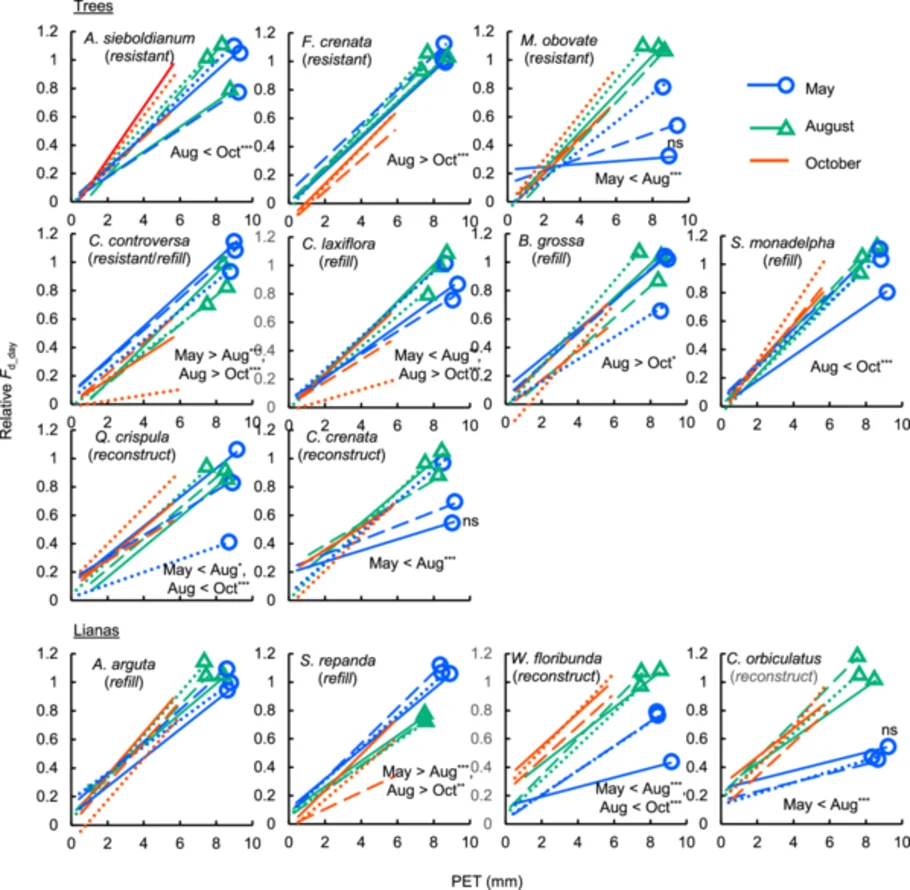
Ordinary least‐squares regressions between daily sap flux (*F*_d_day_), expressed relative to the mean of the ten highest sap flux records for each individual during the monitoring period, and potential evapotranspiration (PET) in cool‐temperate trees and lianas. Three plants were monitored for each species, and data from May, August and October were analysed separately for each individual. Lines of the same type (solid, dashed or dotted) represent the same individual plant, with colours indicating different months. All regressions were significant (*p* < 0.05) except those marked “ns”. Effects of month (May and October relative to August) were tested using a linear mixed‐effects model (see text), *p < 0.05, **p < 0.01, ***p < 0.001. [Color figure can be viewed at wileyonlinelibrary.com]

Aside from these seasonal patterns, the relationship between *F*_d_day_ and PET tended to plateau more noticeably in lianas than in trees during summer (Figure S6), indicating tighter stomatal control in lianas under increasing evaporative demand.

### 3.4 Hydraulic Strategies of Cool‐Temperate Trees and Lianas

In four tree species with relatively small vessels, *K*_s_ did not significantly decrease in late winter compared to summer (*A. sieboldianum*, *F. crenata*, *M. obovata* and *C. controversa*, Figures 1 and 2). These species were recognised to be “*resistant*” to freeze–thaw embolism. In early spring, positive xylem pressure that exceeded tens of kilopascals was observed in four tree species (*C. controversa*, *C. laxiflora*, *B. grossa* and *S. monadelpha*) and two liana species (*A. arguta* and *S. repanda*) (Figures 4a and S7); in most cases, the measured pressure was high enough to potentially push water up to the height of the uppermost or nearly uppermost canopy (Figure 4b). Aside from a *resistant* species *C. controversa*, the species with positive xylem pressure corresponded to those that recovered *K*_s_ by May after it had declined during winter (Figure 2). These species were assumed to '*refill*' their embolised vessels using positive xylem pressure in early spring. In the remaining two tree species (*Q. crispula* and *C. crenata*) and two liana species (*W. floribunda* and *C. orbiculatus*), *K*_s_ was mostly lost in winter and remained low in May (Figure 2). Water conduction during the growing season occurred predominantly through current‐year xylem (Figure S2). Thus, these species were considered to '*reconstruct*' the hydraulic pathway using newly produced vessels in spring.

**Figure 4:**
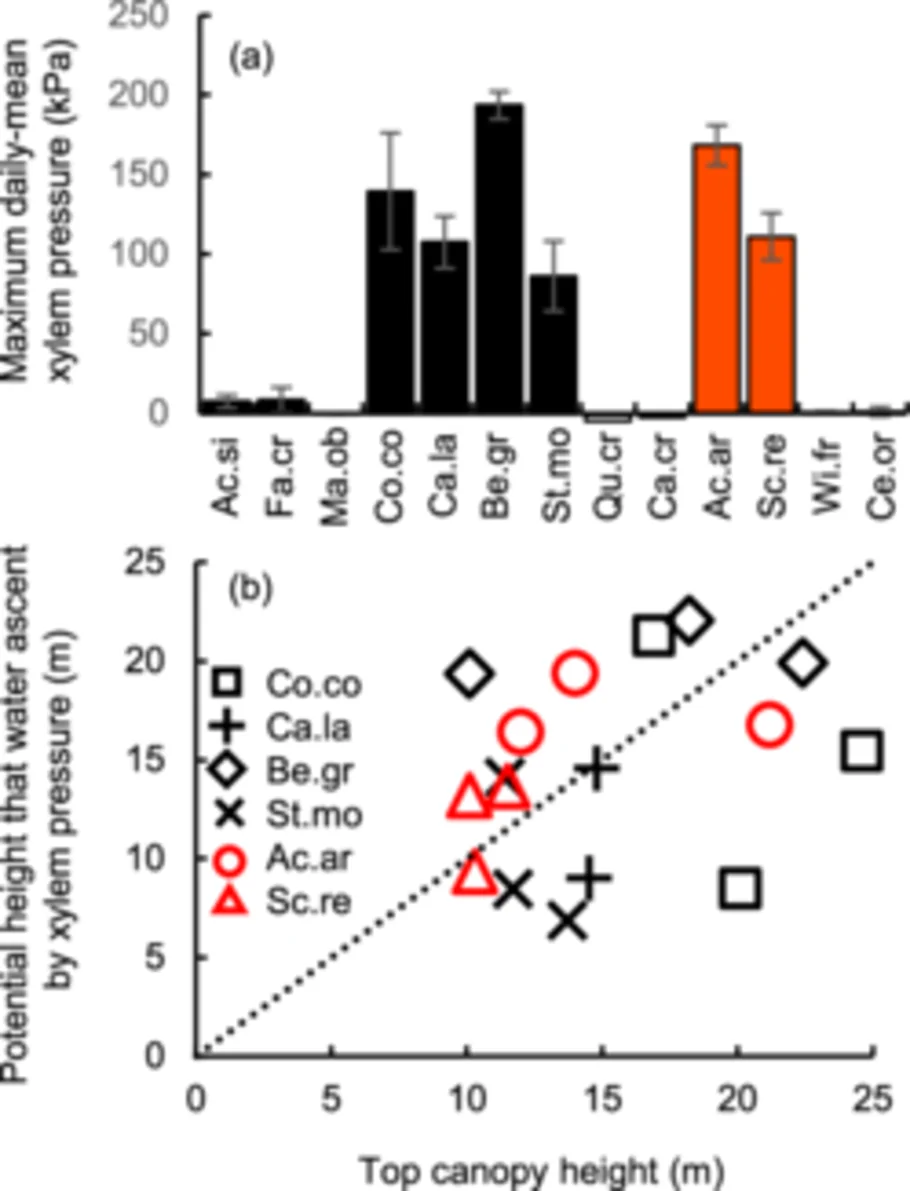
Maximum daily mean xylem pressure measured at the stem base in early spring for cool‐temperate trees and lianas (a), and the relationship between the potential height of water transport estimated from xylem pressure and canopy height in species exhibiting substantial xylem pressure (b). Species names are abbreviated using the first two letters of both the genus and specific epithet. The dotted line indicates the 1:1 relationship (*y* = *x*). Error bars indicate standard errors. *n* = 2 for *F. crenata* and *C. laxiflora*, and *n* = 3 for all other species. [Color figure can be viewed at wileyonlinelibrary.com]

Across all tree and liana species, *D*_h_ was positively related to both *K*_s_ in summer (Figure 5a) and the percentage loss of *K*_s_ (PLC) in winter (Figure 5b). In the winter, PLC increased abruptly for *D*_h_ values below 60 μm and reached nearly 100% for those with larger *D*_h_. Within this safety–efficiency spectrum, tree species were arranged in the order *resistant*, *refill* and *reconstruct* types, from safer to more efficient. Lianas, both *refill* and *reconstruct*, occupied positions beyond the range of trees at the high‐efficiency end of the spectrum while exhibiting PLC values close to 100%.

**Figure 5:**
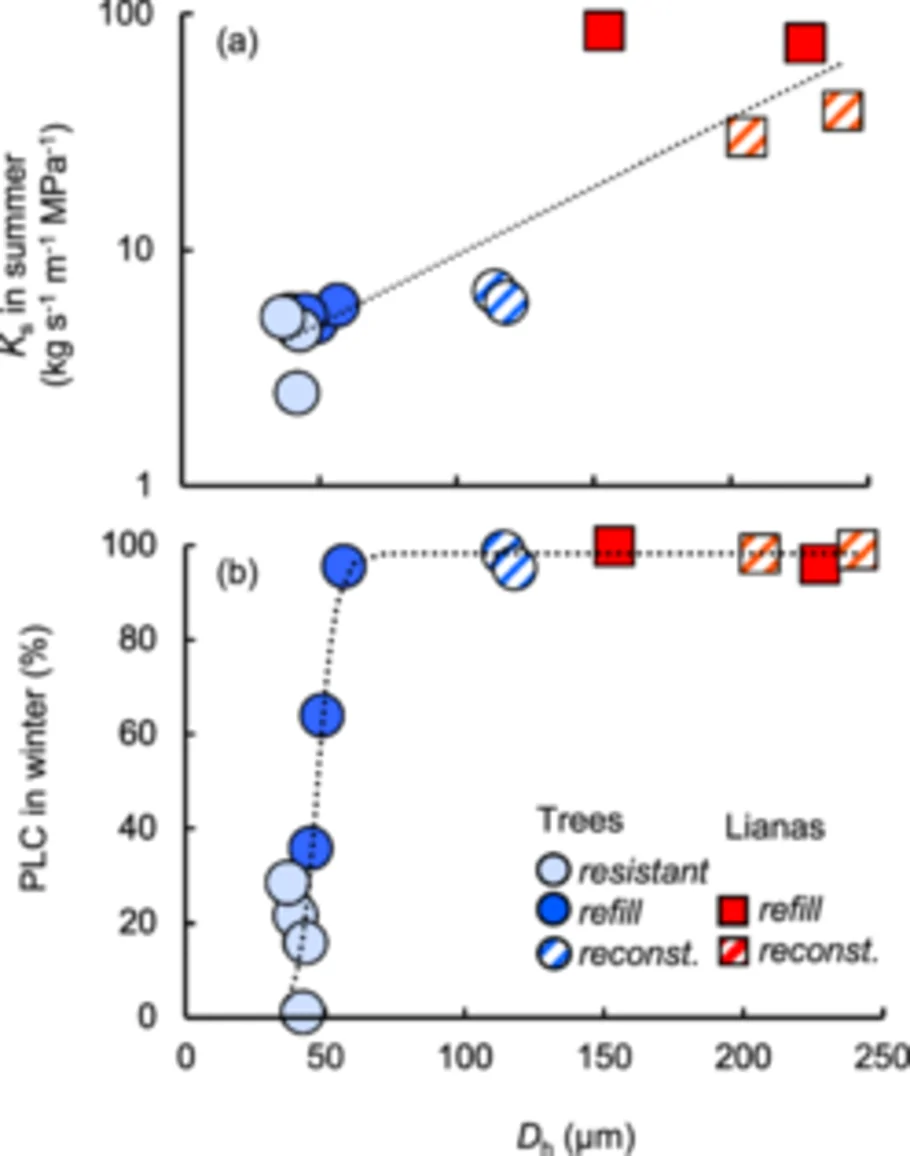
Relationships of xylem area‐specific conductivity (*K*_s_) in summer (a) and percentage loss of conductivity (PLC) in winter relative to summer (b) with hydraulically weighted mean vessel diameter (*D*_h_) in distal stem segments of cool‐temperate trees and lianas. Species are distinguished according to their strategies against freeze‐thaw embolism. Species means were calculated from measurements of five stems. Dotted lines indicate nonlinear least‐squares regressions and are shown to visualise overall trends. [Color figure can be viewed at wileyonlinelibrary.com]

## 4 Discussion

Contrary to the traditional view, the hydraulic function of cool‐temperate lianas did not appear to be disadvantaged by cold winters relative to co‐occurring trees. As predicted by the freeze‐vulnerability hypothesis, lianas possessed very wide vessels and almost completely lost stem hydraulic conductivity during winter. However, the function was fully restored before or during the period of leaf expansion. This recovery was achieved either through *refill* or *reconstruct* mechanisms similar to those observed in temperate trees. During the growing season, the conductive capacity of liana stems was far greater than that of tree stems and was sufficient to supply water to leaf areas many times larger than those supported by tree stems. Although we could not fully test the aftereffects of winter on whole‐plant transpiration (hypothesis iv), chronic stagnation appears unlikely because liana stems showed no evidence of reduced hydraulic capacity during summer. It should also be noted that the whole‐plant leaf mass of cool‐temperate lianas was greater than that of co‐occurring trees (Ichihashi et al. 2017) and was similar to that of tropical lianas with the same basal diameter (Figure S8). Although a prolonged deficit in water availability, including water conduction issues, could lead plants to reduce canopy leaves (Sperry et al. 2002; Bréda et al. 2006; Saiki et al. 2017), this did not appear to be the case for cool‐temperate lianas. Overall, our results did not support a simple freeze‐vulnerability hypothesis. Instead, the results suggest that the ecological success of cool‐temperate lianas depends strongly on mechanisms that restore stem hydraulic function after winter.

Although this study is the first to clearly demonstrate the overwinter hydraulic strategies of lianas, the generality and variation of these strategies should be explored in future studies. At present, we have observed that four more species among cool‐temperate Japanese lianas exhibited water exudation from cut stems in early spring (*Vitis flexuosa*, *V. coignetiae* [Vitaceae], *Actinidia kolomikta* [Actinidiaceae] and *Hydrangea petiolaris* [Hydarngeaceae]) and two species appeared to rely on current‐year xylem (*Tripterygium regelii* [Celastraceae], and *Toxicodendron orientale* [Anacardiaceae], Figure S2n,o), which suggests *refill* and *reconstruct* strategies, respectively (R. Ichihashi, personal observations). Recovery mechanisms similar to those of the present species may be prevalent in cool‐temperate liana communities.

### 4.1 Hydraulic Strategies of Trees and Lianas Against Freeze‐Thaw Embolism

Among the 13 species examined, four tree species were regarded as *resistant* (Figure 2, *C. controversa* may also be counted as a *refill* species). Generally, this type of tree is known to have narrow and short conduits that are resistant to freeze–thaw embolism (Davis et al. 1999; Pittermann and Sperry 2006; Hacke et al. 2017), and, as a result, trades hydraulic safety for efficiency, leading to less productive structural and physiological features (Reich 2014; McCulloh et al. 2019). Consistent with this general trend, the present *resistant* trees were positioned on the safest side along the safety‐efficiency spectrum observed among the 13 tree and liana species (Figure 5). In this continuum, the abrupt increase in winter PLC within the 30–60 μm *D*_h_ range (Figure 5b) closely resembled patterns reported for North American trees (Davis et al. 1999). None of the four liana species were of the *resistant* type but positioned in the most efficient side beyond the tree range (Figure 2). Previous studies on both tropical and temperate lianas demonstrated that they had a much greater amount of leaves per unit stem basal area (Putz 1983; Gerwing and Farias 2000) or above‐ground plant biomass (Wyka et al. 2013; Ichihashi and Tateno 2015; van der Heijden et al. 2015) than co‐occurring trees. By taking advantage of the leafy aboveground structure, as well as their preferential biomass allocation toward stem elongation rather than thickening, lianas attain characteristically high productivity and competitive abilities (Ichihashi and Tateno 2015; Schnitzer and van der Heijden 2019). Thus, lianas operating within this ecological framework could hardly adopt the safe but inefficient *resistant* strategy (Gerolamo et al. 2024; Liu et al. 2026), and this difficulty may have contributed to their reduced abundance and diversity in cold sites. Nevertheless, some liana species, including many root climbers, are known to be less competitive and persist in shady habitats (Ichihashi and Tateno 2011), and possess relatively small vessels (Kusakabe et al. 2025). *Resistant* lianas, if there are any, may be found among such liana species.

Nearly half of the tree species (including the *resistant C. controversa*) and liana species examined generated obvious xylem pressure in early spring and were recognised as *refill* species (Figures 2 and 4). Although the mechanism of xylem pressure generation is not fully understood and could vary depending on the species, it likely involves physical factors, including thermodynamic changes in sap and gas within wood tissue accompanied by freeze–thaw cycles (Graf et al. 2015; Schenk et al. 2021) and/or osmotic forces caused by the loading of solutes, such as sugars, into xylem sap (Ameglio et al. 2004; Westhoff et al. 2008; Schenk et al. 2021). When comparing the pressure profiles between present lianas and trees, the time course and magnitude of xylem pressure were not noticeably different from each other (Figure S7). However, a potentially important difference lay in the size of vessels to be refilled; liana vessels were four times wider (Figure 1a), far exceeding the size range of those typically refilled in trees (McCulloh et al. 2010; Buttó et al. 2021). The overall vessel‐lumen area relative to xylem area was also larger in lianas (Figure S5c), and the extent of xylem embolism prior to refilling was generally more severe in lianas (Figures 2 and 5). Thus, lianas need to restore a substantially larger volume of embolized spaces than trees both at the individual‐vessel and entire‐xylem scales, which likely affects the refilling processes (Brodersen et al. 2010; Knipfer et al. 2016). Whether such differences in xylem anatomy and hydraulic status impose additional hydraulic, mechanical, or metabolic requirements on lianas is currently unknown and represents an important question in studies of refilling mechanisms in temperate lianas and trees.

The remaining two tree species and two liana species were of the *reconstruct* type. In both lianas and trees, the delayed spring recovery of conductivity (Figure 2) and an associated delay of the onset of transpiration (Figure 3) characterised the *reconstruct* species. Similar delays were occasionally documented in this type of ring‐porous trees, which were assumed to reduce the risk of severe damage to new vessels from late frost (Sperry et al. 1994; Hacke and Sauter 1996; Wang et al. 2022). The delay in active transpiration in the present species in May appeared to be less consistently expressed among individuals within each species than the delayed recovery of stem *K*_s_ during the same period. Because *K*_s_ and sap flux were measured on different individuals and were not always obtained in the same years, the weaker delay signal in active transpiration probably reflects natural variations in the timing of vessel and leaf development among individuals and between years. The *reconstruct* strategy is generally associated with substantially wider earlywood vessels in tree species, which compensate for the limited area of functional xylem (McCulloh et al. 2010; Buttó et al. 2021). The present *reconstruct* trees conformed to this pattern (Figure 1a,b). Among lianas, however, vessel diameters of *reconstruct* species were broadly comparable to those of *refill* species. Consequently, the limited conductive area associated with reliance on current‐year xylem (Figure S2) was not offset by greater conduit efficiency, and stem *K*_s_ tended to be lower in *reconstruct* than in *refill* lianas. Nevertheless, this reduction was only relative to *refill* lianas; *reconstruct* lianas still exhibited considerably greater *K*_s_ than co‐occurring trees and maintained *K*_l_ values comparable to those of trees. The ecological significance of these differences in *reconstruct* strategies between lianas and trees remains unclear and warrants further investigation.

### 4.2 Comparison with Previous Studies on Liana Water Relations

The present *K*_s_ and associated parameters were compared with those in several previous studies, most of which were conducted in tropical regions, that examined a number of co‐occurring trees and lianas (details in Note S1, Gartner et al. 1990; Jiménez‐Castillo and Lusk 2013; van der Sande et al. 2019; Zhang et al. 2019; De Guzman et al. 2021; Medina‐Vega et al. 2021; Liu et al. 2026). As a result, the *K*_s_ of the present trees (5.13 ± 0.39 kg s^−1^ m^−1^ MPa^−1^, mean ± SE) were close to values reported in previous studies, whereas those for present lianas (56.7 ± 13.3 kg s^−1^ m^−1^ MPa^−1^) were considerably higher than in most of the previous studies (4–20 kg s^−1^ m^−1^ MPa^−1^). Whether the large difference in reported *K*_s_ between the present and previous studies reflects functional differences among lianas across habitats remains to be determined.

In summer, lianas exhibited a sap flux pattern that suggested tighter stomatal control under higher evaporative demand (Figure S5). A similar yet more pronounced tendency has been frequently reported in tropical lianas (Phillips et al. 1999; Andrade et al. 2005; Chen et al. 2015) and likely reflects a strategy to avoid hydraulic damage from drought rather than a freezing‐related response.

In South American evergreen communities, lianas with narrower vessels occurred in colder regions, suggesting strong selective pressure on vessel size (Lobos‐Catalán and Jiménez‐Castillo 2023), but such selection appeared weaker in deciduous lianas in Japan, despite their occurrence in colder areas (Kusakabe et al. 2023, 2025). For evergreen plants, a more pronounced hydraulic dysfunction caused by larger vessels may directly impair plant performance (Taneda and Tateno 2005), whereas winter hydraulic status may have little effect on the performance of deciduous plants provided that hydraulic function is restored by the start of the growing season, a view supported by our findings. This contrast likely underlies both the differing patterns reported in the two studies of temperate lianas and the tendency for temperate deciduous trees to possess larger vessels than evergreen species (Gorsuch et al. 2001; Kowalski and Wyka 2025).

## 5 Conclusion

We demonstrated that the hydraulic function of cool‐temperate lianas was not inferior to that of co‐occurring trees because both growth forms shared mechanisms for recovering hydraulic function after winter declines under subfreezing conditions. Our results do not support a simple freeze‐vulnerability hypothesis but instead suggest that the ecological success of cool‐temperate lianas depends strongly on mechanisms that restore embolised xylem function. Although this study is the first to clearly demonstrate the overwinter hydraulic strategies of lianas, our conclusions are based on a limited number of species from a single geographic region, and the generality and diversity of these strategies remain to be explored. To date, few studies have examined or even discussed mechanisms underlying the latitudinal distribution patterns of lianas beyond their large vessel size and associated vulnerability to freeze‐thaw embolism. Given the substantial influence of lianas on forest dynamics and ecosystem functioning, greater attention should be directed towards temperate liana communities, which may play an important role in shaping the future dynamics of temperate forests.

## Conflicts of Interest

The authors declare no conflicts of interest.

## Supporting information


**Figure S1:** Meteorological data of the study site through the study period.
**Figure S2:** Photographs of the transverse stem sections of the present trees and lianas after applying a dye solution.
**Figure S3:** Comparison of summer stem *K*_s_ between native and experimentally refilled conditions.
**Figure S4:** Schematic illustrations and photographs of the measurement system for xylem pressure.
**Figure S5:** Results of the vessel‐related anatomical parameters.
**Figure S6:** Analysis of the degree of level‐off in the *F*_d_day_–PET relationships in summer.
**Figure S7:** Xylem pressure charts.
**Figure S8:** Comparison of the published allometric relationships between leaf mass and basal diameter for cool‐temperate and tropical lianas.

**Table S1:** List of the species examined in the present study.
**Table S2:** Comparisons of the mean values of hydraulic and anatomical parameters between trees and lianas.
**Note S1:** Comparison of stem hydraulic parameters of lianas between the present and previous studies.

**Data sheet S1:** Data of hydraulic conductivity and anatomy.

**Data sheet S2:** Data of sup flux.

**Data sheet S3:** Data of xylem pressure.

## Data Availability

All data are provided as Supporting Information (Data Sheets S1–S3).
