# Supplementary material for: Hydraulic Strategies of Cool‐Temperate Trees and Lianas Against Freeze–Thaw Embolism: Is Cold Winter More Severe on Lianas?
Source: Plant Cell Environ. 2026 Sep 17;49(12):10269–79. doi: 10.1111/pce.70889 (PMC13725252; doi:10.1111/pce.70889)
Supplement: Supplementary file 1 [file PCE-49-10269-s002.docx]

| Species | Family | Leaf expansion^†^ | Leaf senescence^†^ |
| --- | --- | --- | --- |
| Trees |  |  |  |
| *Acer sieboldianum* Miq. | Sapindaceae | 30 Apr | 5 Nov |
| *Fagus crenata* Blume | Fagaceae | 26 Apr | 28 Oct |
| *Magnolia obovate* Thunb. | Magnoliaceae | 13 May | 30 Oct |
| *Cornus controversa* Hemsl.var. *controversa* | Cornaceae | 20 Apr | 2 Nov |
| *Betula grossa* Siebold et Zucc. | Batulaceae | 3 May | 4 Nov |
| *Stewartia monadelpha* Siebold et Zucc. | Theaceae | 25 Apr | 8 Nov |
| *Carpinus laxiflora* (Siebold et Zucc.) Blume | Betulaceae | 28 Apr | 27 Oct |
| *Quercus crispula* Blume var. *crispula* | Fagaceae | 5 May | 10 Nov |
| *Castanea crenata* Siebold et Zucc. | Fagaceae | 10 May | 2 Nov |
| Lianas |  |  |  |
| *Actinidia arguta* (Siebold et Zucc.) Planch. ex Miq. var. *arguta* | Actinidiaceae | 22 Apr | 3 Nov |
| *Schisandra repanda* (Siebold et Zucc.) Radlk. | Schisandraceae | 19 Apr | 2 Nov |
| *Wisteria floribunda* (Willd.) DC. | Fabaceae | 8 May | 20 Nov |
| *Celastrus orbiculatus* Thunb. var. *orbiculatus* | Celastraceae | 4 May | 7 Nov |

**Table S1** Species examined in this study

^†^represents the third day after sap flow diurnal cycles became apparent in spring (leaf expansion), and the day they became inapparent in autumn (leaf senescence); means are calculated for three plants per species.

Table S2 Mean ± standard errors of stem anatomical and hydraulic parameters are shown: hydraulically weighted mean vessel diameter (*D*_h_); density of vessels that theoretically accounted for 95% of *K*_­s_ (VD); fraction of vessel lumen in the xylem area; xylem area-specific conductivity (*K*_­s_); percentage loss of *K*_­s_ in winter relative to that in summer (PLC), Huber value (HV) and leaf area-specific conductivity (*K*_l_). Mean values for tree and liana species were compared using Welch’s *t*-test.

|  | *D*_h_  (μm) | VD  (mm^-2^) | Vessel lumen fraction | *K*_­s_  (kg s^-1^ m^-1^ MPa^-1^) | PLC (%) | HV  (×10^-5^ m^2^ m^-2^) | *K*_l_  (×10^-3^ kg s^-1^ m^-1^ MPa^-1^) |
| --- | --- | --- | --- | --- | --- | --- | --- |
| Trees (*n* = 9) | 60.4 ± 10.1 | 81 ± 18 | 0.153 ± 0.015 | 5.13 ± 0.37 | 50.6 ± 12.0 | 12.9 ± 0.6 | 6.41 ± 0.42 |
| Lianas (*n* = 4) | 207 ± 17 | 9.4 ± 2.1 | 0.296 ± 0.013 | 56.7 ± 11.5 | 98.1 ± 0.7 | 2.36 ± 0.25 | 10.9 ± 0.19 |
| *t* | 6.70 | 3.72 | 6.36 | 3.89 | 3.73 | 16.3 | 2.07 |
| *P* | <0.001 | 0.003 | <0.001 | 0.015 | 0.003 | <0.001 | 0.06 |

Note S1. Comparison of stem hydraulic parameters of lianas with previous studies.

The mean specific conductivity (*K*_­s_ ± standard errors) in summer was 56.7 ±13.3 for lianas and 5.13 ± 0.39 kg s^-1^ m^-1^ MPa^-1^ for trees (Fig. 1, Table S2). Compared with seven previous studies on lianas and co-occurring trees (mostly from tropical regions; see Figure below), the *K*_­s_ of our trees was roughly average. In contrast, the *K*_­s_ of our lianas was substantially higher than in five of these studies, except for the one conducted in Mexico (Gartner *et al.*, 1990). Among the previous studies, two from Panama (van der Sande *et al.*, 2013; De Guzman *et al*., 2017) reported leaf area-specific conductivity (*K*_l_) values that were comparable to or even greater than those of our cool-temperate liana species. The ratio of *K*_l_ to *K*_­s_ indicated that the sample branches measured in these two studies had, on average, 5–10 times greater Huber values (HV) than those in our samples. Medina-Vega *et al*. (2021) reported even higher HVs for their lianas, although these values were determined from different branches than those used for *K*_­s_ measurements. Another notable difference was observed in vessel size. Liana species in tropical China examined by Zhang *et al*. (2019) and those in subtropical China examined by Liu et al. (2026) generally had small vessels (< 70 μm, based on the mean of the largest 20 vessel diameters, and 76 ± 13 μm, calculated as hydraulically weighted diameter, respectively) compared with our sample (150–250 μm, calculated as hydraulically weighted mean diameter; Fig. S4).

It remains unclear whether the large variation in hydraulic parameters among studies reflects functional differences of lianas in their respective biomes or resulted primarily from differences in measurement procedures. In the latter case, such discrepancies may arise when the relative position of the measured stem segments along the plant axis differed among studies. Within the same plant, *K*_­s_ tends to be larger in more proximal, thicker, and older stem positions, although it is uncertain whether HV changes similarly along the axis.

The variation in measured *K*_­s_ for lianas critically influences conclusions about whether—and to what extent—hydraulic characteristics differ between co-occurring lianas and trees, which is the focus of all the studies compared here. This issue is therefore important and should be clarified in future research.

Figure. Specific conductivity, (*K*_s_, a), and, where available, leaf area-specific conductivity (*K*_l_, b), and Huber value (c), for liana and tree species examined in this study and in several previous studies. Values (mean ± standard error) were either given in respective papers or calculated from associated data sheets. Numbers of species (*n*) are shown in parentheses on bars in (a) as (*n* liana, *n* tree). Studies 1 (this study) and 2 were conducted in temperate forests (1: Japan; 2: Chile), 3–7 were conducted in tropical forests (3: China; 4: Mexico; 5–7: Panama), and 8 was conducted in subtropical forest in China.

^†^calculated from the ratio of *K*_l_ to *K*_s_

^‡^determined for different branches than those used for *K*_s_ measurement

**Reference**

**De Guzman ME, Santiago LS, Schnitzer SA, Alvarez-Cansino L. 2017.** Trade-offs between water transport capacity and drought resistance in neotropical canopy liana and tree species. *Tree Physiolosy* 37: 1404–1414.

**Gartner BL, Bullock SH, Mooney HA, Brown VB, Whitbeck JL. 1990.** Water transport-properties of vine and tree stems in a tropical deciduous forest. *American Journal of Botany* 77: 742–749.

**Jiménez‐Castillo M, Lusk CH. 2013.** Vascular performance of woody plants in a temperate rain forest: lianas suffer higher levels of freeze–thaw embolism than associated trees. *Functional Ecology* 27: 403–412

**Liu, X., P. Liao, X. Li, Z. Hou, Z. G. Compson, Q. Song, . . . J. Liu. 2026.** Lianas and trees exhibit distinct hydraulic and functional traits in a subtropical forest. *Functional Ecology* 40: 280-293.

**Medina‐Vega JA, Bongers F, Poorter L, Schnitzer SA, Sterck FJ. 2021.** Lianas have more acquisitive traits than trees in a dry but not in a wet forest. *Journal of Ecology* 109: 2367–2384.

**van der Sande MT, Poorter L, Schnitzer SA, Markesteijn L. 2013.** Are lianas more drought-tolerant than trees? A test for the role of hydraulic architecture and other stem and leaf traits. *Oecologia* 172: 961–972.

**Zhang L, Chen Y, Ma K, Bongers F, Sterck FJ. 2019.** Fully exposed canopy tree and liana branches in a tropical forest differ in mechanical traits but are similar in hydraulic traits. *Tree Physiology* 39: 1713–1724.
